# Laser chimeras as a paradigm for multistable patterns in complex systems

**DOI:** 10.1038/ncomms8752

**Published:** 2015-07-14

**Authors:** Laurent Larger, Bogdan Penkovsky, Yuri Maistrenko

**Affiliations:** 1 Institut FEMTO-ST, UMR 6174, Université Bourgogne Franche-Comté, CNRS, 15B avenue des Montboucons, 25030 Besançon, France; 2 Institute of Mathematics, Center for Medical and Biotechnical Research, NAS of Ukraine, Tereschenkivska Street 3, 01601 Kyiv, Ukraine

## Abstract

A chimera state is a rich and fascinating class of self-organized solutions developed in high-dimensional networks. Necessary features of the network for the emergence of such complex but structured motions are non-local and symmetry breaking coupling. An accurate understanding of chimera states is expected to bring important insights on deterministic mechanism occurring in many structurally similar high-dimensional dynamics such as living systems, brain operation principles and even turbulence in hydrodynamics. Here we report on a powerful and highly controllable experiment based on an optoelectronic delayed feedback applied to a wavelength tuneable semiconductor laser, with which a wide variety of chimera patterns can be accurately investigated and interpreted. We uncover a cascade of higher-order chimeras as a pattern transition from *N* to *N*+1 clusters of chaoticity. Finally, we follow visually, as the gain increases, how chimera state is gradually destroyed on the way to apparent turbulence-like system behaviour.

## Abstract (web summary)

Chimera states are a class of self-organized solutions of high-dimensional networks with non-local and symmetry breaking coupling. Here the authors study the chimera patterns generated in a non-linear optical setup and uncover the transition between chimera orders as a pattern across clusters of chaoticity.

## Graphical abstract

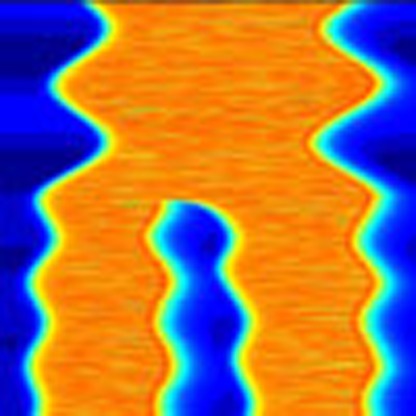

The phenomenon now known as chimera states^1^ was first reported by Kuramoto^2^ for non-local but finite range coupling within a uniform spatial distribution of identical oscillators. It was described as the unexpected spontaneous emergence of spatial clusters of motions, the oscillator dynamics being ‘congruent' within a cluster, but ‘incongruent' from one cluster to another. In the particular case of a network of Kuramoto phase oscillators, the corresponding model was involving a nonlocal coupling function *G*(*x*−*x*′) between oscillators at positions *x* and *x*′:


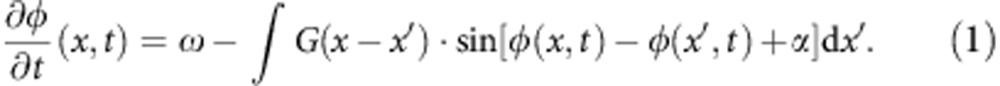


Similar chimera states have been explored for a discrete spatial distribution of phase oscillators around a ring^3^. Such a model has however raised the issue of the unstable character of these states when a finite number *N* of coupled oscillators is concerned (the coupling strength with the 2*R* nearest neighbours along the ring being simplified as constant^4^):


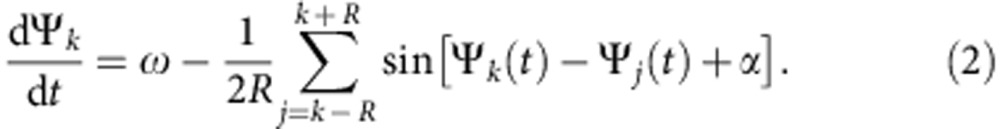


An exponential time duration of this transient with respect to the network size was nevertheless derived^5^. Since space-continuous distributed model as described by equation (1) has an infinite number of oscillators, researchers had the hope to observe real life chimera states in such an experimental configuration, due to the expected infinite transient time. This was indeed reported independently by the two groups^6^,^7^ with spatio-temporal dynamics in the transverse optical intensity distribution of a light beam, and in the spatial concentration of reagents within a chemical reactor, respectively. Since then, several other experiments have reported chimera motions, from a network of mechanically coupled pendula^8^,^9^, and even from purely temporal delay dynamics^10^ as reported by our group. Chimera states currently attracts a growing attention, as illustrated in a recent review by Panaggio and Abrams^11^.

In this article, we report the experimental study of chimera dynamics for the first time in an optoelectronic system, using a delayed feedback tuneable semiconductor laser and involving an Airy non-linear function from a Fabry–Pérot (FP) resonator. In particular, because of the flexibility of the system used, we are able to observe a number of new features of chimera dynamics such as multistable coexistence of so-called multiple-headed chimera states. This experiment is also expected to suggest further studies on chimera states in another well-known delayed feedback semiconductor laser experiment, for example, in the external cavity laser setup^12^. From a more theoretical point of view, we also extend the space–time analogy of delay system^13^ through a rigorous analytic calculation highlighting obvious correspondence with the original model explored by Kuramoto. In this calculation, a signal theory approach of the delayed feedback can suggest one to rewrite the delay dynamics in the form of a convolution product. We additionally report on the identification of specific dynamical features exhibited by delay-based chimera states, among which one finds a quantitatively explored bifurcation scenario between multistable chimera states, and a qualitatively observed transition scenario of the chimera states through turbulence-like dynamics.

## Results

### Delay dynamics requirements for chimera

A delay differential equation (DDE) is a purely temporal dynamics, performing however in an infinite dimensional phase space^14^,^15^,^16^. This infinite phase space is also the feature of the spatio-temporal dynamics, which were of concern in the first experimental demonstrations of chimera state. The common scalar form of such a DDE^17^,^18^ can be given by an equation 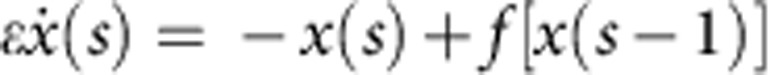, where *s* is the time variable normalized to the delay, and *f* represents a non-linear transformation of the amplitude variable *x*. Though being scalar and of a first-order only, such a dynamic is infinite dimension: the initial conditions required to uniquely define a solution take the form of a functional of time defined over a time delay interval, namely *x*_0_(*s*) with *s*∈[−1; 0]. In the case of a large physical delay (meaning ɛ<<1), the strong multiple timescale character allows for high-complexity phase space^19^.

The space–time analogy of delay systems consists then to represent the dynamics as a discrete time evolution of a functional trajectory 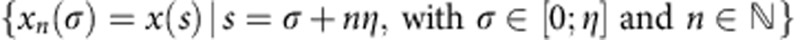. This results in a emulated ‘spatial' dimension of continuously coupled amplitudes, which space spans over a finite ‘virtual space' interval [0; *η*] (with *η*=1+*γ* and *γ*=O(*ɛ*)). The dynamics of this functional trajectory appear as a discrete iteration from *n* to (*n*+1), such a unit iteration corresponding to a time step *η* close to one time delay. The emulated space refers thus to a fast timescale *σ*∈[0; *η*] with a spatial ‘granularity' of the order of *ɛ*, whereas the discrete time variable *n* refers to a long timescale counting roughly the number of time delays.

In delay systems, a chimera is thus expected to appear as a rich multi-clustered pattern over the emulated virtual space, represented by the functional *x*_*n*_(*σ*). This chimera pattern is self-sustained as *n* is growing^10^. However, in its simplest scalar form, a DDE does not allow for such sustained patterns as *n* increases, since they are known to be Lyapunov unstable at any *ɛ*>0 (ref. 20): after some transient, the space is rapidly filled by a unipolar amplitude^21^. We recently showed^10^ that introducing a slow integral term to the DDE:


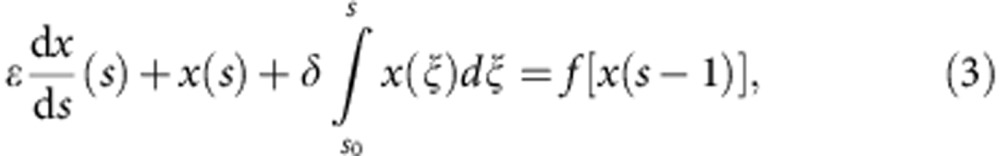


allows for pattern stabilization thus giving rise to robust chimera states. Sustained long-lived patterns *x*_*n*_(*σ*) are obtained over the emulated space *σ* as *n* is iterated.

Additional requirement for obtaining chimera concerns also the function *f*, which associated map (*x*_*n*+1_=*f*[*x*_*n*_]) has to exhibit multistability through a positive feedback at the zero unstable operating point. The unstable central operating point has to connect two asymmetric extrema of the function *f*, a broad minimum for *x*<0 leading to a stable equilibrium, and a sharp maximum for *x*>0 leading to an unstable fixed point, around which chaos can be obtained from the associated map.

**Figure 1:**
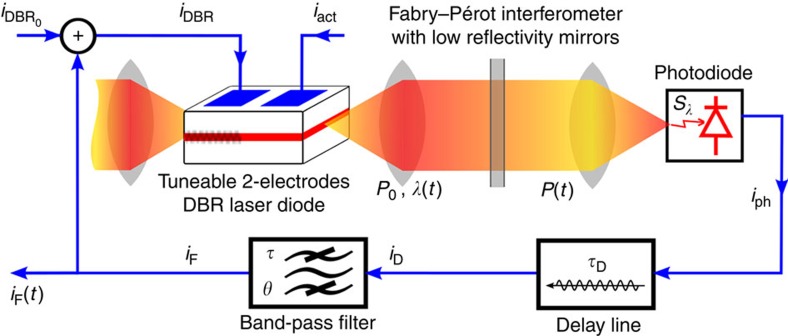
Setup of the laser wavelength dynamics. A tuneable semiconductor laser setup is subjected to a non-linear delayed optoelectronic feedback allowing for highly controllable multiple head chimera states.

Rewriting the integro-differential delay equation into a more common form, one obtains the following two coupled first-order delay equations:


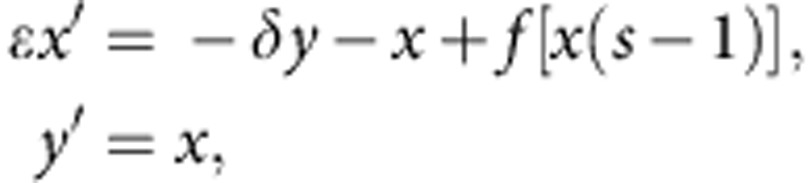


where the additional slow variable *y* accounts for the added integral term. The weight of this additional variable is controlled by the new parameter *δ*>0. This formulation of two coupled differential equation allows to interpret the delay dynamics in terms of simplified slow–fast two-dimensional dynamics^10^,^22^. Neither this formulation nor the integro-differential one could yet help to reveal in a rigorous analytical way the spatio-temporal character of a delay dynamics. To succeed in such an analytical derivation of the spatio-temporal character of a DDE, we propose to adopt an approach inspired by signal theory, which involves a convolution product with a so-called filter impulse response *h*(*s*): *x*(*s*)=∫*h*(*s*−*ξ*)*f*[*x*(*ξ*−1)]d*ξ*. In this integral formulation of a solution *x*(*s*) of the delay dynamics, *h*(*s*) originates from the linear left-hand side of equation (3). It is defined as the inverse Fourier transform of the linear Fourier frequency filtering function. The non-linear delay dynamics is thus revealed as a feedback loop oscillator as in Fig. 1, in which a linear filter provides the argument *x* for the non-linear function *f*, the output of which is delayed, and serves then as the filter input. This ‘convolution product' formulation allows now for a straightforward space–time analogy. Rewriting it with the objective to reveal the discrete functional trajectory *x*_*n*_(*σ*), one obtains (see Supplementary Note 5):


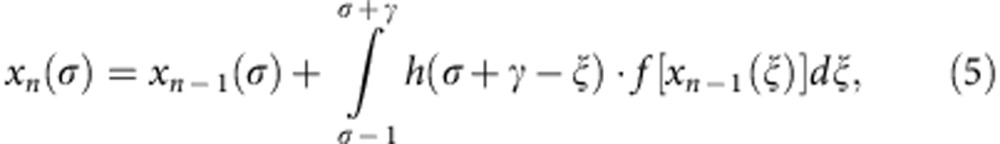


which shows the mapping dynamics from *x*_*n*−1_ to *x*_*n*_, via a spatial non-linear and non-local coupling between the position *σ* and a *ξ* shift around it. When comparing equation (5) with the standard models of equations (1) or (2), 2 originally used to explore chimera states, one can identify *f* as the sine non-linear function coupling the spatially distributed oscillators. The impulse response *h* is also revealed as a distance-dependent coupling weight, appearing as the analogue of the function *G* in equation (1). The shape of the impulse response *h* can be used to identify the typical important parameters in chimera states: the coupling strength is the height of *h*(*s*), and the coupling distance corresponds to the width of this pulse-shaped function (see Supplementary Note 2 for details).

### Laser wavelength delay dynamics

The photonic setup is depicted in Fig. 1. It is designed according to the above described requirements allowing for the observation of chimera in a delay dynamics. The physics and photonic concepts are inspired by a wavelength chaos generator designed previously for optical chaos communication^23^. Two essential modifications have been necessary compared with the original wavelength chaos setup to allow for the emergence of chimera: a band-pass filter instead of low-pass one is providing the left-hand side in equation (3), which is responsible for a long-term stabilization of the chimera pattern; and a FP interferometer is involved instead of a birefringent one, providing an asymmetric non-linear Airy function:


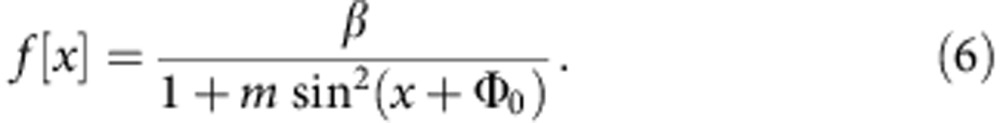


The latter asymmetric function provides the conditions for the coexistence of whether chaotic amplitude oscillations (related to positive amplitudes associated to the sharp maximum of *f*[*x*]), or a constant plateau (at negative amplitudes, related to the broad minimum of *f*[*x*]). The asymmetry is thus at the origin of the different coherent and incoherent clusters of the chimera states.

The operation principles of such an optoelectronic tuneable laser delay oscillator are as follows. A dual-electrode tuneable distributed Bragg reflector (DBR) laser diode provides a laser light beam the colour of which is tuneable around 1.5 μm. The wavelength deviation corresponds in the modelling of equation (4) to the variable *x*, and it is proportional to a tuning electrode current *i*_DBR_. A conventional active electrode of this laser diode receives the injection current *i*_act_ that provides the usual internal gain of the laser, thus setting the actual emitted laser power. An offset current *i*_DBR0_ is added to the tuning electrode, thus allowing to set the central laser wavelength. This offset of the wavelength tuning current is used to set the appropriate parameter Φ_0_ in equation (6), and it is required to ensure a positive feedback condition of the whole delayed-feedback oscillator. Wavelength deviations are then non-linearly converted into intensity ones through the FP, as *x* is scanning back and forth the Airy function from the flat destructive interference condition up to the sharp constructive one. A photodiode converts the optical intensity variations into an electrical signal, which is then delayed in time by *τ*_D_ owing to an easily tuneable electronic delay line. The electronic path of the signal allows for an accurate control of the equation of motion (Equation 3) for the whole delayed optoelectronic oscillator, through an appropriate band-pass filter. This filter has characteristic times *θ* and *τ* that are defining the low and high cutoff frequencies of the filter, respectively. The signal is finally amplified (setting the normalized gain *β* in equation (6)), before being fed back (*i*_F_) onto the laser DBR tuning electrode. The electronic filter output *i*_F_ is the monitored time trace proportional to the wavelength deviation, and thus to *x*(*s*). It is the time varying amplitude that is monitored to observe the space–time chimera patterns 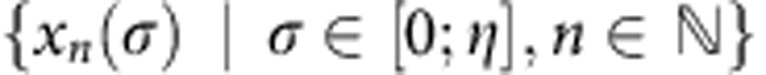. Depending on the setting of the different parameters (offset Φ_0_; gain *β*; and time constants *θ*, *τ* and *τ*_D_), various dynamical regimes of the laser wavelength can be obtained, among which the chimera states discovered in ref. 10 (additional technical and modelling details are provided in Supplementary Notes 1 and 2). The special laser wavelength delay dynamics first proposed in this article allowed to reveal several new features exhibited by these chimera states. These features will be reported in the next sections.

Further analysis and discussion of these phenomena however require normalization (see Supplementary Note 3 for details) of the physical model, especially in terms of timescales. The most important normalized parameters are then defined as the small quantities *ɛ*=*τ*/*τ*_D_ and *δ*=*τ*_D_/*θ* already introduced in equation (3). These two quantities are directly influencing the shape of the impulse response *h*. The parameters *ɛ* and *δ* are indeed controlling the equivalent of the coupling radius and coupling strength, as it was anticipated in the previous section. In the case of concern dealing with a strongly damped band-pass filter dynamics (*θ*≫*τ*), one can even analytically approximate the width of *h*(*s*) as *ɛ*ln(*ɛδ*)^−1^, and its height as *ɛ*^−1^.

### Cascade of multi-headed chimera

**Figure 2:**
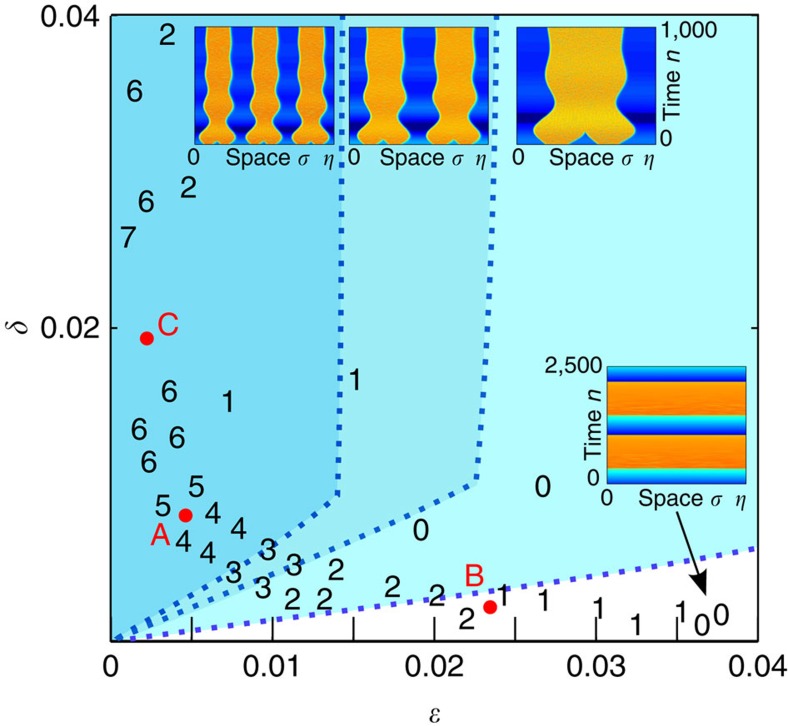
Multistability structure of the chimera dynamics. Colourized regions separated by dotted lines are identified from numerical simulations, and confirmed by the experiment (integer *N* along hyperbola curves, indicating the maximum number of observable chimera heads), with *β*=2.0 and Φ_0_=−0.4. *N*=0 or white region stands for chaotic breather^25^, and otherwise one has *N*-headed chimeras. Crossing a dotted line (numerically determined) from the up left to the low right means a unit decrement for the maximal number *N* of observable chimera heads (any *N*_*σ*_ heads with *N*_*σ*_≤*N* being possible). Insets are examples of space–time plots for *N*=1, 2, 3 (chimeras) and 0 (chaotic breather). They describe the evolution of the delay system trajectory viewed in its space–time representation, the virtual space *σ* being along the horizontal axis, the discrete time *n* along the vertical axis and the amplitude *x*_*n*_(*σ*) being colour encoded. Some reference parameter points are indicated by red dots, *A*=(0.005,0.008), *B*=(0.023,0.0023) and *C*=(0.002,0.019).

**Figure 3:**
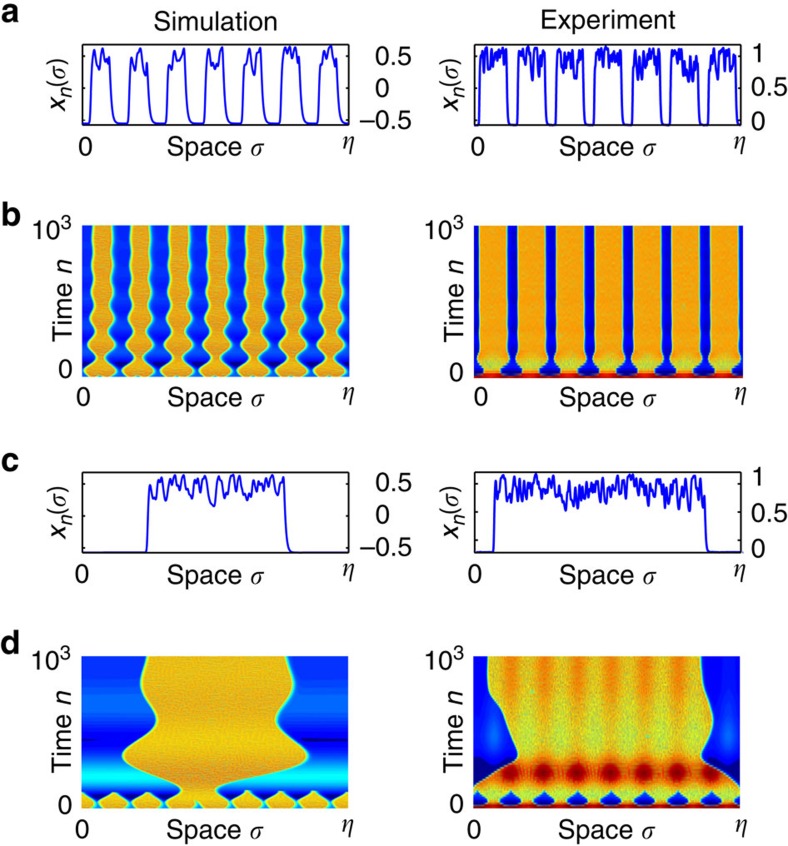
Chimera emergence in space–time representation. Examples of emerging and stabilization of *N*_*σ*_-headed chimera, both from numerics (left plots) and experiment (right plots), at point C in Fig. 2 (*N*_*σ*_=7 and 1 for **a** and **b**, and for **c** and **d**, respectively). (**a**,**c**) Asymptotic (biggest *n*) functional trajectory *x*_*n*_(*σ*). (**b**,**d**) Spatio-temporal pattern birth of *N*_*σ*_-headed chimera. (**b**) Seven-periodic small initial forcing. (**d**) Same as **b** but with eight-periodic small initial forcing; *N*_*σ*_=8 is not stable, thus leading to a single-headed asymptotic chimera state.

As reported in ref. 10, chimera in DDE is revealed as the spontaneous emergence of a particular functional pattern *x*_*n*_(*σ*) showing subintervals over [0;*η*] each of which being characterized whether by a nearly constant negative amplitude, or by a chaotic-like oscillations (see space–time patterns in the in-box of Fig. 2, and time traces in Fig. 3). An amazing peculiarity is that such rich and organized functional behaviour in *σ* can be self-sustained as *n* is iterated. When *N*_*σ*_ chaotic intervals of this kind exist for *σ*∈[0;*η*], the chimera is referred as to a *N*_*σ*_-headed chimera state. A cascade of multi-headed chimera states is established, in a qualitatively comparable way with respect to the Kuramoto model of coupled phase oscillators^24^.

Through the experimental behaviour observed from the laser setup in Fig. 1, as well as from the respective numerical simulations of equation (4), the (*ɛ*,*δ*) parameter space is found to contain a specific multistable bifurcation structure. This structure reveals cascaded regions from the low right to the up left of the (*ɛ*,*δ*) plane, which are successively embedded one in the other with increasing maximal integer *N* of any *N*_*σ*_≤*N* number of possible chimera heads. As shown in Fig. 2, these regions are delimited by bifurcation curves characterized by the transition from *N* to (*N*−1) as *ɛ* is increased, and *δ* is decreased. These regions accumulate with increasing *N* close to the *ɛ*=0^+^ axis. On the opposite side, the lowest value *N*=0 finishes on the *δ*=0^+^ axis. This region does not lead any stable chimera motion, but reveals so-called chaotic breather solutions^25^, a slow envelope alternating fast chaotic oscillations and slow drifts, over time durations of the order of *δ*^−1^. Typical chimera patterns and chaotic breather dynamics are shown in insets of Fig. 2. Numerics (full scan) and experiments (for which a few points only are explored while decreasing *τ*_D_ to scan a hyperbola defined by *ɛδ*=constant) reveal excellent qualitative agreement in the observation of this unusual bifurcation structure. Quantitative discrepancies between numerics and experiments are noticed for the absolute position of the *N*-transition lines, probably related to the influence of noise as well as to uncertain calibration of experimental parameters.

Figure 3 illustrates how a maximum number *N*_*σ*_ is experienced, both in numerics and experiments: for a fixed parameter setting (*ɛ*,*δ*) (point C in Fig. 2) corresponding to *N*=7, one observes that an initial condition imposed as a small sine modulation with seven periods within *η*, indeed leads to the birth of a seven-headed chimera. Trying a small sine modulation with eight periods results in spontaneous switch to the birth of a lower number of heads, which is *N*_*σ*_=1 in the presented case (but any other *N*_*σ*_≤*N*=7 can be observed in principle, even chaotic breather, depending on initial conditions).

**Figure 4:**
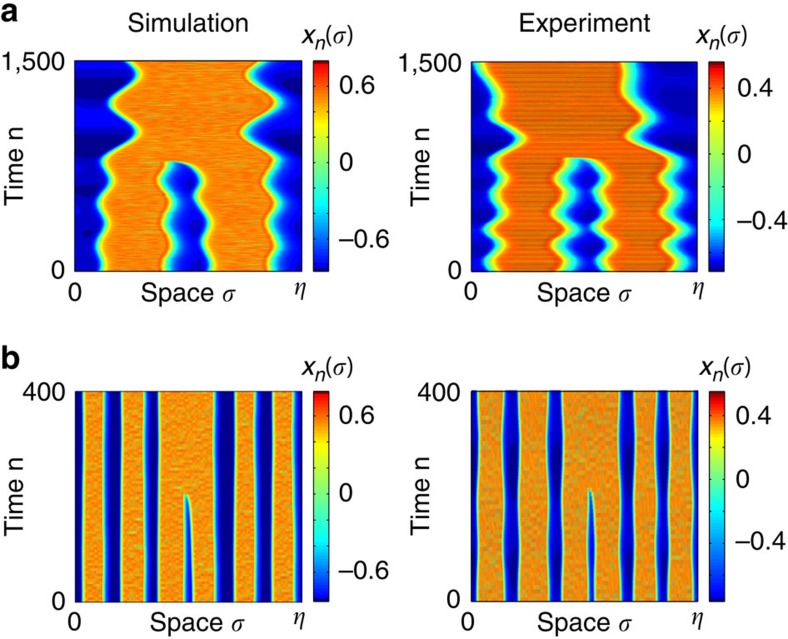
Bifurcation transition at the border of a multistable chimera domain. When starting with a chimera state having the maximum number *N*_*σ*_ of heads in a given domain, the crossing of the boarder towards the next domain with unit decreased maximum number leads to the vanishing of at least one head. Space–time plots show the capture of such bifurcation events, both in numerics and experiment, as *N*-headed chimera becomes unstable, being replaced by (*N*−1) heads. (**a**) 2-to-1 transition (point B in Fig. 2). (**b**) 6-to-5 transition (point A in Fig. 2).

Looking more carefully at the numerical bifurcation lines in Fig. 2, one notices their threshold-like and piecewise linear shape, with a small positive slope close to the origin and then an almost vertical part (strong slope) after a threshold. We anticipate that this peculiar double shape of the bifurcation curves is related to different bifurcation scenarios for the destabilization of the *N*-headed chimera states. Typical *N* to (*N*−1) bifurcation events occurring while crossing such curves, are illustrated in Fig. 4, with captured space–time snapshots for 2-to-1 and 6-to-5 heads transitions. Again, excellent qualitative agreement is found between experiments and numerics. More detailed analysis of this yet unobserved phenomenon is left to a later report.

### Chimera basin

**Figure 5:**
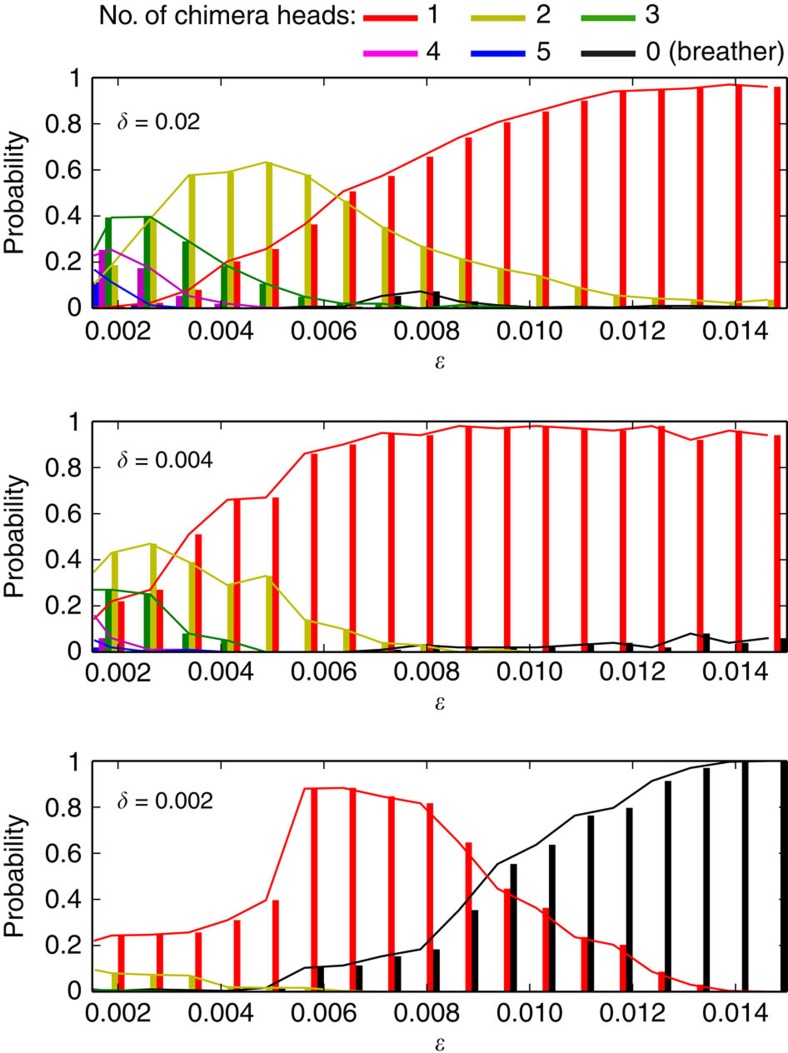
Probability of occurrence for *N*-headed chimera. These probabilities for different number of heads are calculated from the asymptotic *N*-headed chimera states emerged from many different random initial conditions, and for three different values of *δ* (horizontal cut in Fig. 2).

Figure 2a reveals that model (Equation 3) is highly multistable for small and intermediate *ɛ*. This suggests to explore the basins of attraction of the different co-existing attractors. Since time-delayed systems are infinite dimensional through their initial conditions being functional {*x*_0_(*s*)|*s*∈[0,1]}, a precise topological characterization of the basins structure is not directly possible. One can however try to estimate the relative size (measure) of the basins in terms of occurrence probability for each possible solution, after resetting many different random initial conditions. This is illustrated in Fig. 5, which shows the evolution versus *ɛ* of the probability occurrence for *N*_*σ*_-headed chimera for three fixed *δ* values, with *N*_*σ*_=0–5 (0 corresponding to chaotic breather). Each probability has been calculated with 300 different initial noisy conditions *x*_0_(*s*) (uniform amplitude distribution of *x*∈[−1;1]).

In Fig. 5a (*δ*=0.02), for small *ɛ* the most probable solutions are high-order multi-headed chimera, a small fraction only of initial conditions leading to one- or two-headed chimeras. As *ɛ* is increased, *N*_*σ*_=1 and *N*_*σ*_=2 basins are revealing higher and higher occupation of phase space. For an intermediate range of *ɛ*, one notices that two-headed chimera basins reaches a maximum, prevailing on the other possible *N*_*σ*_ with ∼60% of probability of occurrence around *ɛ*=0.005. For larger values of *ɛ*, one-headed chimera basin appears to occupy almost the full explored phase space. In Fig. 5b,c corresponding to smaller *δ* values, qualitatively similar features are observed, except that higher-order chimeras are less and less probable (they would require smaller *ɛ*), and lower *N*_*σ*_ orders predominate together with a growing influence of the chaotic breather (black) as it is visible already in Fig. 2a from the positions of the bifurcation curves.

On the basis of these numerical simulations, we summarize that multi-headed chimeras represent an essential part of the solutions exhibited by equation (4), higher-order chimeras requiring small *ɛ* to predominate. In addition, one could also interestingly connect the coexistence and bifurcation structure of multi-headed chimera states to a recently reported result on optical bit storage through patterns formed using topological solitons^26^.

### Beyond the chimera state

**Figure 6:**
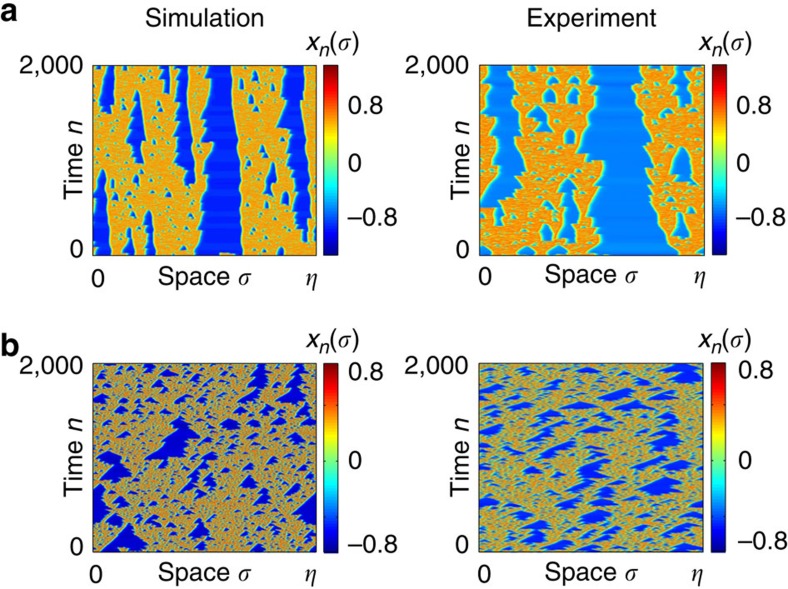
Turbulent destruction of chimera states. Chimera states are being progressively destroyed as parameter *β* is increased, through a turbulent evolution of the chimera pattern (Φ_0_=−0.4, at point A in the (*ɛ*,*δ*) plane of Fig. 2). Turbulence occurs as irregular, more and more frequent, vanishing and appearing events of chimera heads as time *n* is running. (**a**) *β*=2.4. (**b**) *β*=4.0.

The bifurcation diagram (Fig. 2) is obtained for a fixed normalized gain *β*=2.0, providing chimera states as alternated chaotic and quiet amplitudes. Increasing *β* progressively destroys the previously sustained chimera patterns as *n* is iterated. Greater *β* values indeed give rise to a spatio-temporal turbulent-like intermittent behaviour, including both chaotic and quiet amplitudes in an irregular non-sustained manner. This situation is illustrated in Fig. 6 with space–time plots for two higher values of *β*. This transition to turbulence is again nicely consistent between numerics and experiments. One could anticipate, here from the qualitative analogy of the observed patterns, that such progressive vanishing of structured patterns can provide interesting conceptual links with other turbulence phenomena^27^, whether from pipe flow in hydrodynamics, or from a more recently discovered turbulent behaviour in random lasers^28^,^29^. On the contrary, smaller values of *β* transforms progressively the chaotic heads into more regular (periodic) or even quiet plateaus, which situation could have been recently analytically explored^22^,^30^.

## Discussion

Delay equations have always raised difficult and complex issues in many fields such as satellite control, metabolic disorders^14^ and optical chaos^15^. They have also found important practical applications including radar^31^, cryptography^32^,^33^,^34^ and, more recently, brain-inspired computing^35^,^36^,^37^. In the present letter, we have reported on a yet unexplored potential of delay systems in terms of their self-organization capability through a virtual space–time analogy of delay equations, allowing for the interpretation of these complex self-organized motions in terms of chimera states. The reported excellent agreement between numerical simulations of a generic model, and the observed phenomena in a laser-based experiment, suggests the robustness and the intrinsic character of the underlying dynamical concepts. It is also worth noticing in our approach, that delay dynamics intrinsically facilitate the experimental realization of a homogeneous set of coupled oscillator distribution, as required for chimera states. The delay dynamics emulation into coupled oscillators indeed involves the same shared coupling and the same individual oscillator dynamics, as demonstrated by our convolution product description of the delay dynamics and its qualitative analogy with the original model of coupled kuramoto oscillators. We anticipate that such delay systems and their related dynamical phenomena will represent a simple but efficient theoretical tools for investigation of complex self-organized motions, as they are naturally developed in living systems, pattern formation, fluid dynamics phenomena, as well as behaviour in social and technological networks.

## Methods

### Experiments

The acquired signal is corresponding in the setup of Fig. 1, to the output of the band-pass filter limiting the dynamics in the feedback loop. This signal corresponds mathematically to the normalized variable *x*(*t*) in equations (3, 4, 5), and it is physically proportional to the laser wavelength deviation. As described in the setup section, an offset current applied to the DBR tuning electrode of the laser allows to adjust the normalized parameter Φ_0_, thus allowing the selection of the delayed dynamics operation along a positive slope of the FP Airy function. Chimera pattern can then be obtained by increasing gradually from zero the feedback loop gain of the dynamics, which gain is electronically and linearly adjustable via a d.c. voltage applied to an analogue electronic multiplier.

A large memory depth (up to 32 million points) digital Lecroy oscilloscope is used for real-time acquisition of long-time traces covering up to 10,000 time delays. This oscilloscope also provides specific time trace processing capability through short Matlab routines, thus enabling the real-time visualization of the space–time patterns shown in Figs 2, 3, 4 and 6. The main difficulty for this custom real-time chimera pattern observation was to design the adequate algorithm capable for the accurate (10^−5^ required precision) and fast extraction of the spatial width *η*=1+*γ*, for which only the chimera can be clearly observed as a vertical pattern over thousands of time delay duration. This pattern is either strongly tilted in the space–time plane at 10^−4^ precision or even completely invisible for worth precision. The algorithm aims at detecting the most frequent time difference between two plateau-to-chaos sharp transitions in the waveform. Chimera pattern evolution is then analysed while scanning the (*ɛ*,*δ*) plane, which is performed along hyperbola corresponding to constant characteristic times of the band-pass filter *τ* and *θ* (hyperbola equation defined as *ɛδ*=*τ*/*θ*=constant). The hyperbola scan is obtained through the easy electronic tuning of the time delay *τ*_D_ via the increase of a transistor-transistor logic clock frequency *f*_CLK_. This clock indeed controls the speed at which the digitized signal is travelling through the first in first out memory depth used in our digital delay line, the time delay reads then: *τ*_D_=*N*_0_/*f*_CLK_ (where *N*_0_ is a constant integer related to the memory depth of 4,096, and to the small number of sampling periods required by the analogue-to-digital conversion used in the delay line). Increasing *f*_CLK_ remains to scan the hyperbola from the top left to the down right, which leads to decreasing of the maximum number of chimera heads (the highest possible number being forced by adequate initial conditions, on the top left of the hyperbola). Different hyperbolas were scanned through the choice of different *θ* (typically choosing different high-pass cutoff frequencies). The observed chimera pattern in the graphical Matlab window of the oscilloscope allows for the easy detection of the number of heads *N*_*σ*_, or of the chaotic breather solution (0-head), which number can then reported in Fig. 2.

### Numerics

Fourth-order Runge–Kutta integration scheme is used for all the numerical experiments to calculate *x*(*s*), with a fixed time step d*s*=*ɛ*/10. The space–time plots are obtained as in the experiment, through the determination of the duration *η* revealing ‘in average' vertical patterns, stacking vertically the *n*_0_ successive waveforms {*x*(*s*)=*x*_*n*_(*σ*)|*s*=*nη*+*σ* with *σ*∈[0,*η*],*n*=1, 2, … , *n*_0_}.

The procedure for calculating the bifurcation diagram (Fig. 2) is the following. Bifurcation events are detected for several vertical lines in the (*ɛ*,*δ*) plane (constant *ɛ*). For progressively decreasing *δ*, the sustained maximum *N*-headed chimera is tested through the calculation of the dynamics over 10^5^ time delay durations, the solution being initially forced (imposing *x*(*s*) for *s*∈[−1;0]) with a *N*-periods sinusoid. Each *δ* value at which *N* cannot be sustained, whereas *N*−1 can, is used to obtain one point of the *N* to *N*−1 bifurcation curve.

Each chimera basin diagram (Fig. 5) is calculated for 18 values of *ɛ*. The initial interval of [−*η*;0] is interpolated from random initial conditions uniformly distributed within range [−1;1]. For each *ɛ*, 300 numerical experiments is conducted, thus allowing to establish a histogram for the 300 different asymptotic chimera patterns calculated after 10^5^ time delay evolution. Additional technical details about the numerics are provided in Supplementary Note 4.

## Additional information

**How to cite this article:** Larger, L. *et al.* Laser chimeras as a paradigm for multistable patterns in complex systems. *Nat. Commun.* 6:7752 doi: 10.1038/ncomms8752 (2015).

## Supplementary Material

Supplementary Information Supplementary Figures 1-2, Supplementary Notes 1-5 and Supplementary References
