# Supplementary material for: Laser chimeras as a paradigm for multistable patterns in complex systems
Source: Nat Commun. 2015 Jul 14;6:7752. doi: 10.1038/ncomms8752 (PMC4510973; doi:10.1038/ncomms8752)
Supplement: Supplementary Information — Supplementary Figures 1-2, Supplementary Notes 1-5 and Supplementary References [file ncomms8752-s1.pdf]

Supplementary figures

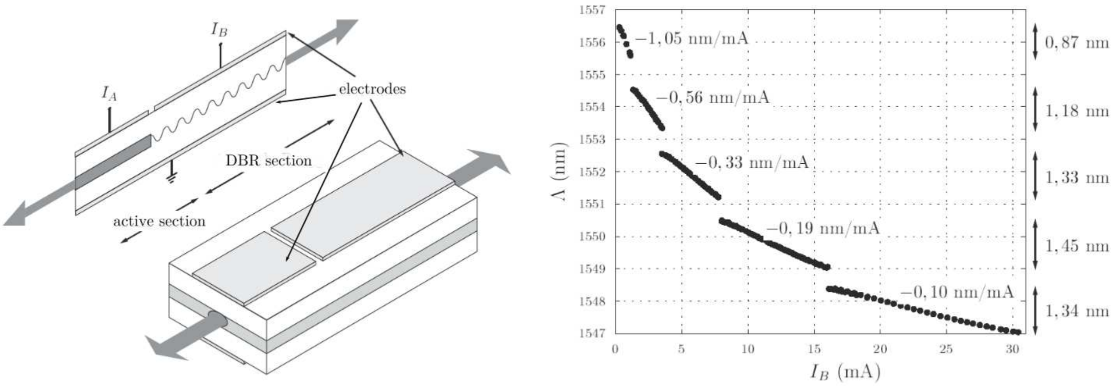

Supplementary Figure 1: The tunable DBR laser. Left: laser structure, with I_A being the current used to adjust the laser output power level, and I_B being the wavelength tuning current. Right: experimental measure of the tuning characteristics vs. I_B, showing the stepwise successive continuous tuning ranges with the corresponding sensitivity S_λ.

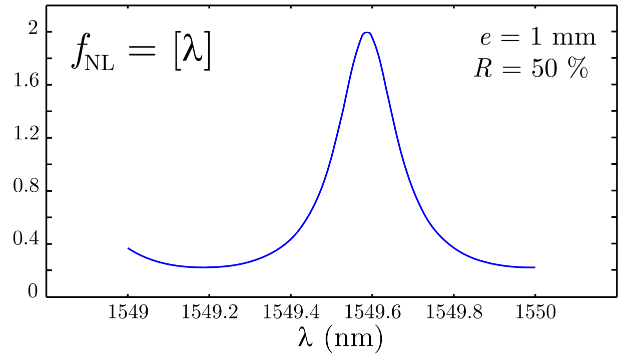

Supplementary Figure 2: Theoretical profile of the wavelength-to-intensity nonlinear conversion provided by a Fabry-Pérot plate.

Supplementary Note 1: Experimental setup

Figure 1 (main article) illustrates schematically the nonlinear delayed feedback loop experiment providing a dynamics on the wavelength of tunable semiconductor laser.

It consists mainly of 5 functional physical elements:

1. The tunable laser diode is expected to provide a laser light beam which wavelength can be continuousy and linearly tuned through an electrical drive current. It is a linear electrical-to-optical converter, more precisely a current-to-laser wavelength converter.

2. The Fabry-Pérot interferometer is ruling the nonlinear transformation occurring from the wavelength deviation, into optical intensity fluctuations. It is a "nonlinear" optical-to-optical converter. Though it is commonly considered as a linear optical filter for an incoming optical wave amplitude, it has to be considered here as nonlinear in our delay dynamics problem, since the input variable is the instantaneous wavelength of laser, and not the electromagnetic field amplitude (the output being the optical intensity).

3. The photodiode is required since we expect to have consistent physical quantities in such a closed feedback architecture: we need to recover back an electrical signal before closing the oscillation loop. This is fulfilled by the photodiode, converting linearly, and instantaneously the incoming optical intensity into a detected photo-current (photodiode bandwidth, as well as laser tuning bandwidth, are far above the characteristic times of the next elements of the electrical feedback, typically beyond 10 MHz).

4. An electrical digital delay line is providing the delay in the feedback, moreover in a very flexible way. The delay can be indeed easily adjusted whether through the depth of the FIFO (First In First Out) memory used to generate the delay, or through the digital clock frequency (0.6 MHz to 40 MHz) ruling the speed at which the signal is travelling through the memory. This bandwidth ($\simeq$ 120 kHz), though not that important, is also assumed to be negligeable (instantaneous delay of any relevant Fourier frequency component of the travelling signal) compared to the last element.

5. An electronic filter is finally used to force the dominant time scales ruling the dynamics of the oscillator loop. The filter is of bandpass type, with the simplest second order profile. A (variable) gain function can also be attached to this last element, allowing for the tuning of the overall closed loop gain of the nonlinear delay oscillator. This variable gain is simply provided by a multiplier circuit, one input being the signal to amplify, and the other being a tunable constant voltage controlling thus linearly the normalized feedback β around the values of interest (typ. from 0 to 5).

Supplementary Note 2: Modeling

The wavelength tunable laser

This element is the central one in a wavelength dynamics implemented through an optoelectronic feedback loop. It allows for a physical variable conversion from an electrical signal (a current) into a laser wavelength deviation. The device is a DBR (distributed Bragg reflector)

multi-electrode laser diode as depicted in Fig. 1. It consists of a Fabry-Pérot cavity having natural reflector on one side, and a tunable DBR reflector on the other side. The latter reflector has a maximum Bragg reflection wavelength determined by the optical periodicity $n\Lambda_B$ of a Bragg reflector, which can be tuned through the change of the refractive index of the Bragg material. This variation is caused by the modulation of the free carrier occupying the diode junction where the Bragg grating is designed, through the modulation of a forward injection current I_B. The lasing wavelength is thus directly tuned according to the maximum of reflection of the DBR grating. The laser gain is provided by a gain section next to (but electrically indepedent from) the Bragg section, through an electrical pumping current I_A (lasing threshold typ. around 8 mA, operating value at 20 mA, leading to a useful lightbeam of ca. 1 mW optical power).

$$\lambda = \lambda_0 + S_\lambda \, I_B \quad \text{or} \quad \nu = \nu_0 + S_\nu \, I_B, \tag{1}$$

For a DBR current modulated around 11 mA ($\lambda_0 \simeq 1549.8$ nm), the device exhibits a continuous tuning range with an efficiency of about $S_\lambda \simeq 0.19$ nm/mA. This continuous tuning range spans over 1.45 nm, thus corresponding to a peak to peak current modulation amplitude of ca. 7 mA (see Fig. 1 Right).
If one assumes the current I_B is delivered by a voltage source $v_N = v_B + \alpha\, v$ in series with a "strong" (Norton model) resistor R_N (much stronger than the static resistance of the forwardly biaised DBR junction), so that the current I_B can be confidently determined as:

$$I_B = \frac{v_N}{R_N} = \frac{v_B}{R_N} + \frac{\alpha\, v}{R_N} =< I_B > + \frac{\alpha\, v}{R_N}, \tag{2}$$

where v_B/R_N is the DC contribution to the junction current bias $< I_B >$ (around 11 mA), such that the other $v-$contribution to I_B verifies $< v >= 0$. α is a voltage-linearly adjustable factor used to tune the overall gain in the oscillator loop.

Notice finally that the laser delivers a variable wavelength light beam, with an optical power P_0. This laser beam is passed then through an optical filter in order to perform a nonlinear transformation of the input wavelength into the output light intensity.

The nonlinear transformation

Contrarily to the original wavelength chaos generator reported in [1], the observation of chimera states requires two important differences. One of these relates to an asymmetric shape of the nonlinear transformation involved in the delay dynamics. With the usual two-wave interference provided e.g. by a birefringent interferometer, one indeed observes symmetric maxima and minima described analytically by the well known $\cos^2$ or $\sin^2$ nonlinear profile.

Since the wavelength delay dynamics involves a nonlinear transformation from a wavelength variation into the intensity output of a spectral optical filter, a rather straightforward solution to obtain asymmetry between minima and maxima is to make use of a multiple wave interferometer, such as a Fabry-Pérot one:

$$f_{\rm NL}[\lambda] = \frac{A}{1 + m\, \sin^2 \Phi} = \frac{A}{1 + m\, \sin^2(2\pi n e/\lambda)} = \frac{P_{\rm FP}}{P_0}, \tag{3}$$

where $2ne$ is the optical path corresponding to one round trip in the cavity, $m = 4R/(1-R)^2$ is a function of the reflection coefficient in intensity R of each mirror end of the cavity influencing

the "contrast" ($[(1+R)/(1-R)]^2$) of the Fabry-Pérot transfer function, $A = 1/(1-R)$ is a vertical scaling factor, and $\Phi(t) = 2\pi ne/\lambda(t)$ is a phase modulated due to the variation of the laser wavelength $\lambda(t) = \lambda_0 + \delta\lambda(t)$. Considering the very small deviation of the wavelength around a central value ($\delta\lambda/\lambda_0 \simeq 10^{-3}$), one could write $\Phi(t) = \Phi_0 + \delta\Phi(t)$, with $\Phi_0 = 2\pi ne/\lambda_0 = 2\pi ne\nu_0/c$ and $\delta\Phi(t) = -(2\pi ne/\lambda_0^2)\cdot\delta\lambda(t) = (2\pi ne\nu_0/c)\cdot\delta\nu(t)$. The fine tuning of the central laser wavelength (through a mean DBR and/or active laser current, and/or through the controlled laser temperature) allows to adjust the actual rest point of the Fabry-Pérot modulation transfer function around which the modulation operates (e.g. positive or negative slope, around a broad minimum or a sharp maximum of the nonlinear function formed by this wavelength-to-intensity conversion through the Fabry-Pérot effect).

One could notice here the conceptual analogy between the FM delay dynamics [2, 3], and the wavelength delay dynamics [1]. The voltage controlled oscillator in the first case corresponds to the tunable laser in the second. The resonant RLC filters which filtering profile determines the nonlinear function in the first case, corresponds to the optical filtering function provided by the two-wave or multiple-wave interference in the second case.

Physical values of the Fabry-Pérot parameters have been properly chosen to exhibit a very few broad resonances and anti-resonances within the wavelength tuning range of the laser. The Fabry-Pérot was designed with a glass plate of $e = 5$ mm thickness, and intensity reflection coefficients $R = 50$ % (20 GHz free spectral range, or 0.16 nm around 1.55 μm).

The Fabry-Pérot output intensity fluctuations are pratically converted into an electrical signal by a simple photodiode, thus providing an electrical signal which can be used for the electronic feedback onto the DBR electrode of the tunable laser:

$$u_{\mathrm{ph}}(t) = R\,S\,P_{\mathrm{FP}}(t), \tag{4}$$

where $S = 0.9$ A/W is the photodiode sensitivity, and R is the equivalent resistor of a typical transimpedance amplifier typically used to convert the photocurrent into a voltage. The product $R\,S$ (in V/W) is then the conversion efficiency of an integrated amplified photodiode.

The delay

The natural physical delay line with an optoelectronic setup is obviously a fiber spool. The magnitude of the delay is however to be compared with the slowest characteristic response time involved in the feedback loop. In the present setup, we are planning to observe dynamics in the μs range, thus implying much greater time delays (up to ms). It appears thus much more practical to design an electronic delay line based on FIFO (First In First Out) memories through which the signal is traveling at a speed determined by a digital clock frequency. With this alternative technical solution for the delay, values of τ_D as high as a few seconds can be achieved in principle, down to ca. 1 ms (changing both the clock frequency f_{CLK} from 40 MHz to 600 kHz, as well as the FIFO depth, from 512 to 65536).

The very simple equation for the (linear) transfer function of a delay line is expressed as follows, in the time domain and frequency domain respectively:

$$u_D(t) = u_{\mathrm{ph}}(t-\tau_D) \quad \xrightarrow{\mathrm{FT}} \quad H_{\mathrm{del}}(\omega) = \frac{U_D(\omega)}{U_{\mathrm{ph}}(\omega)} = e^{-i\omega\,\tau_D}. \tag{5}$$

Special attention needs however to be drawn on the possible response times τ which can be set, due to basic sampling theory limitations (the sampling rate being defined by f_{CLK}, thus imposing a maximum non-zero Fourier component up to $f_{\mathrm{CLK}}/2$).

The Fourier frequency filter ruling the integro-differential dynamics

The motion of the feedback loop oscillator, differently speaking the differential equation ruling the dynamics of the designed nonlinear delayed oscillator, is originating from the limiting Fourier filtering performed by the oscillation loop in the linear regime. More precisely, it has been shown in [2] that observation of virtual chimera motions requires a bandpass filtering, which results in an integro-differential process in terms of equation of motion.

Assuming a bandpass filtering of the simplest form, the corresponding Fourier filter is trivially expressed as:

$$H(\omega) = \frac{V(\omega)}{U_D(\omega)} = \frac{i\omega\,\theta\,G_0}{(1+i\omega\,\theta)(1+i\omega\,\tau)} \xrightarrow{\mathrm{FT}^{-1}} \frac{1}{\theta}\int_{t_0}^{t} v(\xi)\,\mathrm{d}\xi + \Big(1+\frac{\tau}{\theta}\Big)\,v(t) + \tau\,\frac{\mathrm{d}v}{\mathrm{d}t}(t) = G_0\,u_D(t), \tag{6}$$

where $\tau = (2\pi f_h)^{-1}$ is the high cut-off frequency of the corresponding low pass filter, and $\theta = (2\pi f_l)^{-1}$ is the low cut-off frequency of the corresponding high-pass filter, and G_0 is the gain of the filter.

Equation (6)-Left is a Fourier domain second order bandpass filter, which illustrates the global frequency response of the filter (relative phase shift and attenuation between output and input, when a sinusoidal signal is used at the input).
Equation (6)-Right is a local (in time) description through a differential law ruling the evolution of the filter output v at time t, knowing the input signal u_D. The *global* description for v in the time domain can be also expressed, as a convolution (integral) operation involving the so-called impulse reponse of the filter $h(t)$, practically defined as the inverse Fourier transform of the Fourier domain filter transfer function in Eq. (6)-Left:

$$v(t) = \int_{-\infty}^{t} h(t-\xi)u_D(\xi)\,\mathrm{d}\xi \quad \text{with} \quad h(t) = \frac{e^{-t/\theta}}{\tau(1-\tau/\theta)}\left[e^{-t\,(\tau^{-1}-\theta^{-1})} - \frac{\tau}{\theta}\right] h_{\mathrm{H}}(t), \tag{7}$$

where $h_{\mathrm{H}}(t)$ is the stepwise Heavyside function (null for $t<0$ and unity for $t \geq 0$), reflecting the causal feature of any impulse response. The impulse response of the second order bandpass filter can be analyzed as follows:

- At the origine, it has a stepwise variation from zero to τ^{-1}, identically to the first order low pass filter with the same characteristic time τ (low-pass case: $H(\omega) = 1/(1+i\omega\tau)$ and $\mathrm{FT}^{-1}[H(\omega)] = h(t) = \tau^{-1}\,e^{-t/\tau}h_{\mathrm{H}}(t)$);
- From the origine, the function is decreasing linearly with a slope $-\tau^{-2}(1+\tau/\theta)$ (which is steeper than the low pass one, $-\tau^{-2}$);
- It crosses zero at time $t_0 = [\tau \cdot \ln(\theta/\tau)]/(1-\tau/\theta)$;
- It reaches a minimum at $2t_0$, with an amplitude close to $-\theta^{-1}(1-\alpha)$ where α is a small quantity scaling as $2(\tau/\theta)[\ln(\theta/\tau)]/(1-\tau/\theta)$;

- It finally decreases slowly exponentially to zero from negative values as $(-\theta^{-1})e^{-t/\theta}/(1-\tau/\theta)$.

Since $\tau \ll \theta$ in the definition of the bandpass filter, it is worth noticing that $(\tau/\theta) \ll 1$ is a very small quantity (of the order of 10^{-4}).

It is worth noticing here the main difference in terms of impulse response, between a low pass filter delay dynamics (the usual in most of the popular delay dynamics, e.g. Mackey-galss or Ikeda ones), and the bandpass of highest importance for the observation of sustained chimeras patterns. The previous description of the bandpass impulse response reveals non-monotonus feature of its "Mexican hat"-like profile through the negative values it takes, on the contrary to the usual impulse response of the low pass case which is indeed monotonuously decreasing to zero.

Supplementary Note 3: Delay dynamics normalization

A very common way for the time normalization in delay equations is to introduce a new time variable $s = t/\tau_D$ ($\mathrm{d}t = \tau_D\,\mathrm{d}s$) normalized to the delay. The differential equation (6), once re-written in s and combined with the time domain delay process (5), reads as:

$$\delta \int_{s_0}^{s} v(\xi)\,\mathrm{d}\xi + (1+\delta\varepsilon)\,v(s) + \varepsilon\,\frac{\mathrm{d}v}{\mathrm{d}s}(s) = G_0\,u_{\mathrm{ph}}(s-1), \tag{8}$$

where $\delta = \tau_D/\theta$ and $\varepsilon = \tau/\tau_D$ are two (generally small in the large delay and broadband situation of concern) temporal parameters, which are crucial for the stability of the virtual chimera states. This is precisely one of the purpose in the present article. The experimental setup is indeed intended to explore physically the influence of these temporal parameters on the chimera states stability. Numerical simulations as well as analytical investigations, have estimated the practical range of interest for the scanning of different chimera patterns in the experiment.

When combining Eqs. (1) to (4) together with Eq. (8), one is led to the amplitude normalization of the dynamics, considering the various conversion efficiencies and gains in the optoelectronic feedback loop. The unit free variable $x(s)$ can be defined as the varying argument $\delta\Phi$ inside the $\sin^2$ −function appearing in Eq. (3). In order to convert v into $\delta\Phi$ in the left hand side of Eq. (8), one needs to multiply both sides of the differential equation with the constant factor $(\alpha\,S_\lambda/R_N)\cdot(2\pi ne/\lambda_0^2)$ (where $\alpha(U_G) = U_G/U_{G_0}$ is the linearly tuneable gain already described in Eq.(2)) leading to:

$$x = \frac{2\pi ne\,\alpha(U_G)\,S_\lambda}{\lambda_0^2\,R_N}\,v. \tag{9}$$

As a direct consequence, two amplitude parameters are revealed through this normalization, the feedback loop gain β and the offset phase Φ_0 ruling the mean operating point for the Fabry-Pérot nonlinear modulation transfer function,

$$\beta = \frac{2\pi ne\,\alpha(U_G)\,S_\lambda\,RSG_0P_0A}{\lambda_0^2\,R_N} \quad \text{and} \quad \Phi_0 = \frac{2\pi ne}{\lambda_0 + S_\lambda\,v_B/R_N}. \tag{10}$$

One should notice in these last two normalized expressions with respect to physical parameters, that both appear as independently adjustable experimentally:

- β through the voltage controlled factor $\alpha(U_G)$,
- and Φ_0 through the offset voltage v_B.

The normalized dynamics written as a vectorial (2 variables, x and $y = \int x\,\mathrm{d}s$) delay differential equation reads finally as follows:

$$\begin{aligned}\varepsilon\,\frac{\mathrm{d}x}{\mathrm{d}s}(s) &= -(1+\delta\varepsilon)\cdot x(s) - \delta\cdot y(s) + \frac{\beta}{1+m\,\sin^2[x(s-1)+\Phi_0]}, \\ \frac{\mathrm{d}y}{\mathrm{d}s}(s) &= x(s).\end{aligned} \tag{11}$$

One could slightly simplify the previous equation when neglecting the quantity $\delta\varepsilon = \tau/\theta \ll 1$.

Supplementary Note 4: Numerical simulations

The previous normalized model was numerically investigated using a fourth order Runge-Kutta algorithm with constant integration time step, as described in the "Methods" section at the end of the main article.

Despite the fact that experiments obviously showed sustained patterns and thus not transient chimera states (10^6 time delays are indeed corresponding to easily testable duration in practice, with time duration of the order of hours free running experiment with fixed parameter), it is important for the numerical viewpoint to also indicate how long, in terms of number of time delays integration duration, one has to consider the numerics in order to get the asymptotic domains in the $(\varepsilon,\delta)-$plane.
The actually required time for chimeras to emerge spontaneously from random initial conditions, is practically less than 10^2 time delays. Thus, one could in principle choose a calculation slightly longer than this typical duration. For a safe calcultation margin ensuring the removal of any transient, our numerical simulations have systematically considered a duration of 10^5. This calculation duration was used to obtain the borders of the domains reported in Fig. 2 in the main article. Calculation duration values of about 10^3 to 10^4 time delays as in Ref. [1] of the main article are however already enough to check that the obtained solution indeed numerically persists at any point of the tested parameter plane.

One could finally also remark that small quantitative disagreement can be observed in the upper-left part of Fig.2 in the main article. This has to be discussed however with respect to the experimental difficulty to accurately calibrate the parameters in this particular region of the hyperbola curves. Qualitative disagreement however only holds when one consider two neighboring experimental hyperbola curves. When staying on the same hyperbola (as it is actually the case in the experimental scanning conditions), the agreement between numerics and experiments is indeed very good (the sequence of successive number of possible heads). Changing the hyperbola constant (jumping from one hyperbola to another) is unfortunately rather critical experimentally in this upper-left part of the (ε,δ)-plane. The consequence is that the relative positions of the hyperbola might not be controlled with high enough accuracy to get "perfect" agreement. Good qualitative is nevertheless globally obtained with a very good accuracy, confirming that the model is well capturing the experimental observations.

Supplementary Note 5: Analytics highlighting the space-time analogy

The dynamics, instead of a local differential equation as in (6) or (8), can be expressed as a global convolution product as already proposed in Eq. (7) when describing the impulse response $h(t)$ of the loop filtering as the inverse Fourier transform of filter transfer function $H(\omega)$. Considering the dimensionless time unit, one has:

$$x(s) = \int_{-\infty}^{s} h(s-\xi)\cdot f[x(\xi-1)]\,\mathrm{d}\xi. \tag{12}$$

The principle of the space time analogy is based on the mutiple time scale character of the delay dynamics, leading to the definition of the normalized time s as being decomposed into a short time scale contribution of the order of $\varepsilon = \tau/\tau_D$, and into a long time scale contribution of the order of the unit delay:

$$s = n\cdot\eta + \sigma, \qquad \text{with} \qquad \eta = 1+\gamma \simeq 1 \tag{13}$$

where $n \in \mathbb{N}$ counts roughly the number of time delay interval since the origine of time (since $\gamma = \mathrm{o}(\varepsilon) \ll 1$), and $\sigma \in [0;\eta]$ is a small time variable identifying the fine temporal position within one approximate time delay interval. σ is then represented as a virtual space coordinate spanning over a finite virtual space interval, namely $[0;\eta]$.
One can rewrite the convolution product as

$$x(s) = x_n(\sigma) = \int_{-\infty}^{n\eta+\sigma} h(n\eta+\sigma-\xi)\cdot f[x(\xi-1)]\,\mathrm{d}\xi$$

Splitting then the integral domain into two domains, more specifically $]-\infty;(n-1)\eta+\sigma]$, and $[(n-1)\eta+\sigma;n\eta+\sigma]$, one enables an interpretation with a space-time dynamics, consisting in a discrete time map from $n-1$ to n, and a spatially extended coupling within the map model:

$$x_n(\sigma) = x_{n-1}(\sigma) + I_n(\sigma), \tag{14}$$

$$\begin{aligned}
\text{where} \quad x_{n-1}(\sigma) &= \int_{-\infty}^{(n-1)\eta+\sigma} h(s-\xi)\cdot f[x(\xi-1)]\,\mathrm{d}\xi \\
\text{and} \quad I_n(\sigma) &= \int_{(n-1)\eta+\sigma}^{n\eta+\sigma} h(s-\xi)\cdot f[x(\xi-1)]\,\mathrm{d}\xi \quad \text{(with } s \text{ defined as in Eq. (13)).}
\end{aligned}$$

With a simple change of variable $\zeta = \xi+\eta-1-n\eta = \xi+\gamma-n\eta$, one can rewrite the spatial coupling integral as:

$$I_n(\sigma) = \int_{\sigma-1}^{\sigma+\gamma} h(\sigma+\gamma-\zeta)\cdot f[x_{n-1}(\zeta)]\,\mathrm{d}\zeta. \tag{15}$$

With the previous expression for $I_n(\sigma)$, this contribution to the dynamics appears as a non-linear non local "spatial" coupling, according to a nonlinear function defined as $f[x_{n-1}(\zeta)]$, and a "spatially" extended coupling strength with a spatial extension profile determined by the time reversed (due to the convolution integral) impulse response $h(s)$, as discussed for

the modeling issues (Suppl. Note 2). The spatial non locallity depends on the actual spread of the impulse response. The local coupling at σ thus amounts to $1 + h(\gamma)$, and the spatial extension around the central position σ is spread from $(\sigma - \Delta)$ to $(\sigma + \gamma)$, with coupling weights of $h(0)$ and $h(\Delta)$, respectively.
The extension Δ ($\ll 1$, thus fitting within the integral domain from $\sigma - 1$ to $\sigma + \gamma$) is an arbitrary distance (or short time) defined by the features of the chosen $h(s)$, above which the impulse response has a negligeable coupling amplitude. This has been analytically derived in the modeling section (Suppl. Note 2), Eq.(7), for a second order bandpass filter. It was moreover shown there, that such a bandpass filter is responsible for the integral term in the integro-differential delay equation of motion, and thus is responsible for some specific stability features of the positive feedback delay dynamics, compared to the usual “differential-only” delay equation (e.g. Mackey-Glass or Ikeda models). In terms of nonlocal spatial coupling, one realizes that such bandpass feature is equivalent to an increase by a factor of $\ln(\theta/\tau) = -\ln(\varepsilon\delta)$ of the original extension Δ, compared to the differential-only situation.

Supplementary references

[1] Larger, L., Goedgebuer, J.-P. & Merolla, J.-M. Chaotic oscillator in wavelength: A new setup for investigating differential difference equations describing nonlinear dynamics. *IEEE J. Quantum Electron.* **34**, 594–601 (1998).

[2] Larger, L., Penkovskyi, B. & Maistrenko, Y. L. Virtual chimera states for delayed-feedback systems. *Phys. Rev. Lett.* **111**, 054103 (2013).

[3] Larger, L., Udaltsov, V. S. & Goedgebuer, J.-P. Chaotic dynamics of oscillator based on circuits with VCO and nonlinear delayed feedback. *Electron. Lett.* **36**, 199–200 (2000).
